# Effect of an audience on trainee stress and performance during simulated neonatal intubation: a randomized crossover trial

**DOI:** 10.1186/s12909-018-1338-4

**Published:** 2018-10-03

**Authors:** Brahim Bensouda, Romain Mandel, Abdelwaheb Mejri, Jean Lachapelle, Marie St-Hilaire, Nabeel Ali

**Affiliations:** Maisonneuve Rosemont Hospital, Pediatric Department, University of Montreal, 5415 Boulevard de l’Assomption, Montréal, QC H1T2M4 Canada

**Keywords:** Simulation, Performance, Trainee, Audience, Stress

## Abstract

**Background:**

Neonatal intubation is a stressful procedure taught to trainees. This procedure can attract additional observers. The impact of observers on neonatal intubation performance by trainees has not been studied. Our objective was to evaluate if additional observers present during neonatal mannequin endotracheal intubation (NMEI) by junior trainees, affects their performance and their stress levels.

**Methods:**

A randomized cross over trial was conducted. First year residents with no experience in neonatal intubation were assigned to NMEI condition A or B randomly on day 1. Subjects were crossed over to the other condition on day 2.

Condition A: Only one audience member was present Condition B: Presence of an audience of 5 health care providers.

Differences in the time to successful NMEI was recorded and compared between conditions. A portable heart rate monitor was used to measure peak heart rate above baseline during NMEI under both conditions.

**Results:**

Forty nine residents were recruited. 72% were female with a median age of 25 years (IQR: 24–27). Time to successful intubation was comparable under both conditions with a mean difference of − 3.94 s (95% CI: -8.2,0.4). Peak heart rate was significantly lower under condition A (mean difference − 11.9 beats/min, 95% CI -15.98 to − 7.78).

**Conclusion:**

Although the time required to NMEI did not increase, our results suggest that presence of observers significantly increases trainee stress. The addition of extraneous observers during simulation training may better equip residents to deal with such stressors.

**Trial registration:**

Date of registration: March 2016, NCT 02726724.

**Electronic supplementary material:**

The online version of this article (10.1186/s12909-018-1338-4) contains supplementary material, which is available to authorized users.

## Background

Endotracheal intubation (ETI) is a common procedure in neonatal intensive care units (NICU). This procedure is associated with adverse effects such as laryngospasm and subsequent risk of intracranial hemorrhage. Such effects can cause significant neonatal morbidity [1]. Traditionally, residents are often taught neonatal ETI during their first NICU rotation, and any poor delivery room experience can create a lack of confidence [2]. Due to increased trainee numbers, reduced duty hours, increased use of non-invasive ventilation, as well of the presence of other health care professionals competing for a limited number of procedures, opportunities to teach ETI in a clinical setting are dwindling. In order to prepare trainees adequately for their first clinical encounter, simulation is increasingly used. ETI in the NICU must often be performed in the presence of multiple health care workers who may be observing the trainee attempting their initial ETI in a live neonate.

The presence of observers is known to increase stress levels. The Trier Social Stress Test (TSST) is a widely used protocol to induce stress in laboratory settings. In the original TSST, where subjects have to deliver a speech and perform mental arithmetic in front of an audience, there was a significant increase in stress hormones and individual heart rates [3]. Most of the studies using the TSST show heart rate increases of 24 to 34% [4, 5]. It has also been suggested that stress can interfere with the performance of technical skills in critical situations [6]. Currently, simulated ETI or other pediatric procedures are taught to trainees without accounting for the presence of multiple observers. When a trainee is faced with an unfamiliar situation, he will experience it as stressful and this could lead to adverse performance [7]. It is unknown if additional observers could adversely affect performance of trainees. Therefore, we conducted a randomized crossover study to obtain objective evidence that junior trainees are adversely affected by the presence of a large audience during neonatal mannequin endotracheal intubation (NMEI).

### Hypothesis

We hypothesized that the time required to successful NMEI would be negatively impacted by the presence of observers. We also hypothesized that stress levels would be higher in the presence of an audience as measured by the subject heart rate.

## Methods

We conducted a randomized cross over study at Maisonneuve Rosemont Hospital, a Level III NICU in Montreal, Canada. The institutional scientific and ethics review board approved the study. Every participant gave written informed consent before enrolment. Participants were informed of their right to discontinue participation at any time.

### Participants

Residents were recruited during their pediatrics or neonatology rotation in our hospital. All first-year trainees were approached sequentially by the main investigator to participate during the recruitment period. Trainees with prior experience in live newborn intubation were excluded in order to select novice learners. Students with a history of beta blocker use within the past year, and students with a history of antidepressant medication were excluded. All trainees had completed the neonatal resuscitation program (NRP) training course during which they had the chance to practice NMEI on a mannequin at the start of their rotation block. For our study, participants were told they would perform NMEI and that their performance during two NMEI scenarios would be evaluated in the context of scientific investigation. The true goal of the study was not revealed until the end of the experience. Participants were asked to keep their experience confidential.

### Intervention

A full body neonatal mannequin approximating a term newborn (Newborn Anne, Laerdal Medical Corporation, Wappinger Falls, NY) was setup in a hospital delivery room suite on a radiant warmer. Subjects were asked to wear a cardiac monitor, and 5 min later after a period of rest, they were then called to the delivery room. Heart rate was assessed continuously, starting 5 min before NMEI and stopping 5 min after cessation of the procedure, using a wireless heart rate monitor (Polar M400). Five minutes after their arrival to the delivery room, they were given a stylet and a 3.5 ET tube and asked to intubate orally a neonatal mannequin. No practice attempts were allowed. After 30 s, the residents were reminded verbally that they had 15 remaining seconds. Each individual attempt was recorded as the time in seconds from insertion to the removal of the laryngoscope, using a chronometer operated by the main investigator. Success or failure of the attempt was assessed by the investigator after removal of the laryngoscope. If the resident failed the intubation, he was given the laryngoscope and the tube for a subsequent attempt and the chronometer was restarted from the time it was stopped. The duration of all attempts was limited to a total of 45 s. A participant who never succeeded, was assigned an intubation time of 45 s. A maximum time limit of 45 s was chosen, to best approximate the physiological tolerance of a live infant. The NRP recommends limiting each attempt to 30 s. However, previous studies suggest a mean intubation time of 38 s in trainees [8].

In this randomized crossover study, each volunteer participated in two NMEI scenarios under two different conditions, A and B, and served as his/her own control. The sequence, A then B, or B then A, was assigned randomly.

#### Condition a

Only one staff neonatologist was present with the participant during NMEI.

#### Condition B

An audience of 5 people were present to watch the participant during NMEI, with at least 2 clearly identified neonatologists, one being the main investigator. The other 3 observers were health care professionals (nurses or other trainees) from the perinatal service. Audience members stood within 2 m of the subject, but remained silent, neutral and did not interact with the trainee.

The residents who performed NMEI in condition A on day one were crossed-over to NMEI the next day under condition B, and vice versa. The time and the location of the 2 procedures were the same.

### Primary outcome

We chose time to successful intubation as the primary outcome. We did not use success or failure of intubation as the primary outcome, since we expected all trainees to eventually be able to intubate a static mannequin with no vital signs given enough time. The mean difference in total intubation time between condition A and B was compared.

### Secondary outcomes

The increase in heart rate from baseline (5 min before NMEI) to peak (during laryngoscopy) as a percentage value was calculated for each subject, and the mean difference between condition A and B was compared.

The rate of success on first attempt as well as the rate of success across all attempts was reported under each condition. Successful intubation was defined as tube placement below the vocal cords within 45 s.

### Sample size

To estimate the required sample size, we used the study by O’Donnel et al. who gave an approximate intubation time for residents of 38 s in live infants, with a standard deviation of 20 s [8]. Using a two tailed alpha threshold of 0.05 and power of 80%, a sample size of 50 subjects was estimated to detect an 8 s difference time between the two different groups. Eight seconds represents a 25% change in time to intubate and is clinically significant for a newborn.

### Randomization

A simple randomization scheme was used, and the sequence of envelopes was generated by a random coin flipper (www.random.org). Allocation was revealed by opening a pre-prepared sealed envelope before participants were called to the delivery room. Neither the participants nor the investigator was blinded to allocation. The audience present under condition B did not know if the resident had already performed under condition A.

### Statistics

The difference in NMEI time and heart rate increase was calculated between conditions A and B for each candidate, and the mean difference within subjects was compared to the null value of 0 using a two tailed paired t-test. The rate of success of intubation between both conditions was compared with the McNemar chi-squared test. An alpha level of 0.05 was considered significant. All calculations were done with SAS 9.4 (Toronto, ON).

## Results

**Fig. 1:**
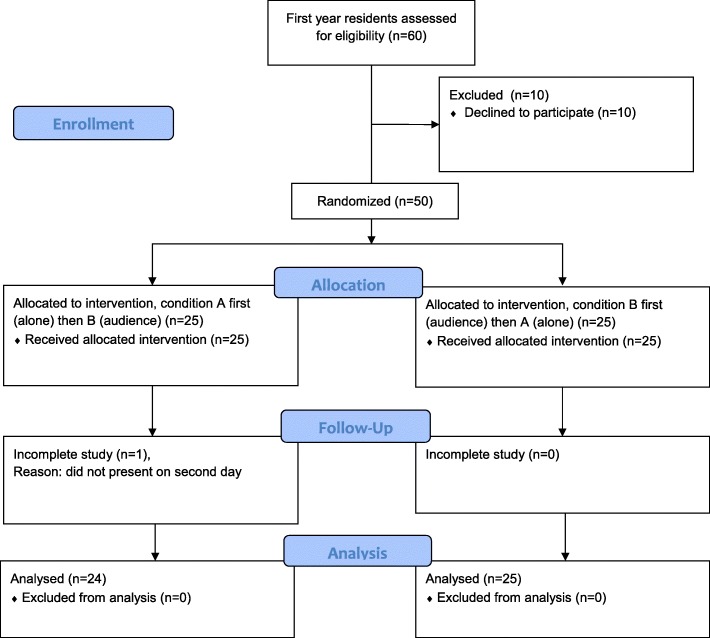
Participant Flow

Subjects were recruited from November 2015 to November 2016. Participant flow is shown in Fig. 1. Of the 60 eligible first year residents in the period, 10 declined or did not respond to our request to participate. One participant completed condition A but never presented for condition B, and no outcome data is available for this subject. Median age for all participants was 25 years (IQR 24–27) and 72% were female.

Results for all 49 analyzed participants are presented in Table 1.

**Table 1:** Performance during intubation under conditions A and B

	Condition A (staff only)	Condition B (audience)	Difference A-B	*p*-value
Time to intubation (sec), mean (95% CI)	30.9 (27.7, 34.1)	34.8 (31.7, 37.9)	−3.94 (− 8.2, 0.4)	*0.07* ^*1*^
% heart rate increase over baseline (bpm), mean (95% CI)	38.4 (34.6, 42.2)	50.3 (45.3, 55.3)	−11.9 (−15.98, −7.78)	*< 0.001* ^*1*^
Number of successful intubations for all attempts (%)	39 (79.6)	32 (65.3)	–	*0.14* ^*2*^
Number of successful intubations on first attempt (%)	31 (63.2)	29 (59.2)	–	*0.83* ^*2*^

*bpm* beats per minute, *CI* confidence interval, ^1^paired t-test, ^2^McNemar test

There was no significant difference in the time to intubation under condition A and B. However, under condition B, the increase in heart rate over baseline was significantly higher than condition A. The rate of successful intubation on first attempt, or all attempts was comparable.

Due to the crossover design, additional analyses were performed to control for sequence. To test for carryover effects, the effect of sequence was compared (AB vs BA) with no significant difference either in time to intubation (*p* = 0.09) or heart rate (*p* = 0.79).

## Discussion

This is the first study in the medical area which evaluated the impact of an audience on performance during simulated intubation, in junior trainees. The time and the rate of successful NMEI was identical under both conditions, whether with one or multiple observers. However, we documented that a larger audience alone, neutral and without other distractors was associated with increased heart rate, a possible indicator of increased stress. There are many definitions of stress. The World Health Organization defined work-related stress as the response people may have when presented with work demands and pressures that are matched to their knowledge and abilities and which challenge their ability to cope. The relationship between stress and performance is not linear. Stress can have different effect on individuals. Studies have demonstrated that the effects of stress intensity on behavior are characterized by an inverted-U-shaped function while low or high levels of stress lead to performance impairment on tests of vigilance and working memory, a moderate level of stress leads to performance improvement [9]. It was also reported that baseline stress in human participants measurements were predictive of individual resilience to stress, including the impact stress had on physiological reactivity and performance [10]. The response to acute stress is highly dependent on the individual’s perception of demands and resources [11]. Research has found that psychological and social resources may be most protective at relatively low or moderate levels of stress and less so at high levels of stress [12].

Increased stress level does not always affect performance in the medical domain as was reported by many investigators. In a neonatal simulation procedure when both subjective and objective measures of stress increased over the duration of the simulated experience, there was no association between performance and either cortisol or subjective stress [13]. However, in a simulated trauma scenario, some aspects of performance and immediate recall appear to be impaired in complex clinical scenarios in which they exhibit elevated subjective and physiologic stress responses [14]. The impact of stress on performance may vary with the trainee’s perception, the task at hand and the ability to cope.

The presence of an audience has been described as increasing stress in the literature. In a psycho-social study with 183 participants going through the TSST who experienced either an unsupportive audience, a supportive audience, or no audience, both audience conditions produced significantly higher cortisol, heart rate, and blood pressure responses to the stressful tasks, relative to the no-audience control [15]. Belletier and al provided evidence that simply being watched by evaluative others leads individuals to perform poorly and to choke on a classic measure of executive control [16].

Two different processes leading to poor performance in high-pressure situations were described in the literature. The first process, choking, occurs because worries distract executive attention. In the second, the pressure shifts too much executive attention toward the task at hand, which may cause poor performance in routine (non-attention-demanding) tasks relying on skill processes and procedures that normally run best outside of conscious awareness [16]. Choking under pressure is in situations where one is being watched by others especially when one’s performance is being evaluated [16, 17].

A study conducted in thirty physicians who performed two simulated resuscitation scenarios in random order, one scenario without additional distractors and one scenario with additional distractors (noise, scripted family member) shows that the performance scores were lower under experimental conditions than under control conditions. External distractors markedly reduced the quality of cardiopulmonary resuscitation and there were no overall significant differences in median performance scores between groups with different levels of training (1st/2nd year residents, 4th/5th year residents: consultant anaesthetists, *p* = 0.519) [18]. Hunziker and al investigated the influence of a short task focusing strategy on perceived stress levels and performance of rescuers in a simulated CPR scenario [19]. In this randomized-controlled trial, a total of 124 volunteer medical students were randomized to receive a 10-min instruction to cope with stress by loudly posing two task-focusing questions (“what is the patient’s condition?”, “what immediate action is needed?”) when feeling overwhelmed by stress (intervention group) or a control group. A brief stress-coping strategy moderately decreased perceived stress without significantly affecting performance in a simulated CPR [19]. The presence of family members, which is a particular kind of audience, during procedures, has been studied in recent literature. In a retrospective study, the presence of family members did not have an impact on the success of pediatric intubation [20]. However, junior residents are not in favor of the presence of family because they are worried about being judged by the parents [21, 22].

Residents are faced with stressful procedures in the NICU and they are afraid of being judged by multiple observers, whose presence is often unnecessary, and by the attending staff responsible for their evaluation. The present study suggests another mechanism of stress in the junior resident. By understanding mechanisms of stress, medical educators might improve training by providing learners with resources in stress management. The repetition of stressful events can lead to depression, burnout and anxiety [23].

This study has several limitations. The ETI is done on a neonatal mannequin and does not necessarily represent real life. There were no distractors around the procedure and the results may have been different if there was a combination of an audience and distractors, as would be the case in a real NICU setting. In particular, there were no vital signs given to subjects to simulate patient bradycardia and desaturation during attempts. Our study was powered to detect a change in time to intubation of 8 s, based on the mean time to intubation of 38 s in O’Donnel et al. study done in real infants [8]. The mean duration in our study was 30 s under condition A, which is 20% shorter than expected. This would reduce the minimal effect size we could detect. Our study was not powered to detect a difference in intubation success rates, though our success rate of 65 and 79% in condition A and B was much higher than rates 20% reported in live infant [24]. This suggests a lower difficulty level for NMEI versus live ETI, particularly since attempt durations were not limited by physiologic tolerance. Heart rate measurements were a secondary outcome of our study, since our sample size was calculated based on estimated time to intubation. Furthermore, heart rate is one of several measures of stress, and we did not look at others measures, such as blood pressure or salivary cortisol.

## Conclusion

The presence of an external audience did not negatively impact the duration of simulated intubation by novice trainees, but the presence of non-mandatory personnel as observers is stressful to trainees. Our study has important implications for simulation training in neonatal ETI, and possibly in training for other stressful procedures. To adequately prepare trainees to manage stress, it is imperative that simulation scenarios be representative of real life clinical situations where multiple observers are often present.

## Additional files

Additional file 1: Raw Data. Measurements taken for each participant during the simulated intubation (XLSX 46 kb)

Additional file 2: Statistical analysis. Analysis of categorical and continuous variables by condition and sequence (DOCX 17 kb)

## Abbreviations

CPR: Cardiopulmonary resuscitation
ETI: Endotracheal intubation
NEMI: Neonatal endotracheal mannequin intubation
NICU: Neonatal intensive care unit
TSST: The Trier Social Stress Test
