# Supplementary material for: Effect of an audience on trainee stress and performance during simulated neonatal intubation: a randomized crossover trial
Source: BMC Med Educ. 2018 Oct 3;18:230. doi: 10.1186/s12909-018-1338-4 (PMC6171149; doi:10.1186/s12909-018-1338-4)
Supplement: Supplementary file 2 — Statistical analysis. Analysis of categorical and continuous variables by condition and sequence (DOCX 17 kb) [file 12909_2018_1338_MOESM2_ESM.docx]

49 of 50 residents completed the study. Median age was 25 ans (IQR : 24-27). 72% female.

1. **Analysis 1 : Comparison s of outcome by protocol design**

**Table1. Comparison of outcomes continuous data (by protocol)**

| **Outcome** | **Situation A : no observer** | | **Situation B : multiple observers** | | **Difference A-B** | | **pvalue** |
| --- | --- | --- | --- | --- | --- | --- | --- |
|  | **mean** | **deviation standard** | **man** | **deviation standard** | **mean** | **deviation standard** |  |
| Time in seconds | 26.90 | 9.93 | 29.09 | 9.76 | -1.78 | 14.11 | 0.5184 |
| Temps in seconds 45 | 30.88 | 11.57 | 34.82 | 10.94 | -3.94 | 15.40 | 0.0765 |
| Absolute change in HR | 31.28 | 10.11 | 39.53 | 11.82 | -8.27 | 11.62 | <.0001 |
| Percentage change in HR | 38.39 | 13.57 | 50.28 | 17.75 | -11.88 | 14.77 | <.0001 |
| Tube position | 10.08 | 1.55 | 11.36 | 1.95 | 0.26 | 0.91 | 0.2021 |
| *parametric paired ttest for normal data and nonparametric wilcoxon test for non normal data | | | | |  |  |  |

1. **Analysis 2 : Comparison dof outcomes continues controlling for crossover design**

**2.1 Outcome 1 : time to Intubation**

| *Type 3 Tests of Fixed Effects* | | | | |
| --- | --- | --- | --- | --- |
| *Effect* | *Num DF* | *Den DF* | *F Value* | *Pr > F* |
| *sequence* | 1 | 67 | 2.94 | 0.0909 |
| *Condition* | 1 | 67 | 1.13 | 0.2911 |

| *Least Squares Means* | | | |
| --- | --- | --- | --- |
| *Effet* | *categorie* | *Estimate* | *Standard Error* |
| *sequence* | AB | 29.9 | 1.5 |
| *sequence* | BA | 26.1 | 1.6 |
| *Condition* | Condition A: no observer | 26.8 | 1.5 |
| *Condition* | Conidition B multiple observer | 29.1 | 1.7 |

| *Differences of Least Squares Means* | | | | | | |
| --- | --- | --- | --- | --- | --- | --- |
| *Effect* | *Difference* | *Differences of Least Squares Means* | *Standard Error* | *DF* | *t Value* | *Pr > \|t\|* |
| *sequence* | AB -BA | 3.82 | 2.23 | 67 | 1.72 | 0.0909 |
| *Condition* | Condition A: sans observateur - Conidition B avec observateur | -2.37 | 2.23 | 67 | -1.06 | 0.2911 |

**2.1 Outcome 2 : time to Intubation (45 secs for failure)**

| *Type 3 Tests of Fixed Effects* | | | | |
| --- | --- | --- | --- | --- |
| *Effect* | *Num DF* | *Den DF* | *F Value* | *Pr > F* |
| *sequence* | 1 | 47 | 0.07 | 0.7903 |
| *Condition* | 1 | 47 | 3.03 | 0.0884 |

| *Least Squares Means* | | | |
| --- | --- | --- | --- |
| *Effect* | *categorie* | *Estimate* | *Standard Error* |
| *sequence* | AB | 32.9348 | 1.7078 |
| *sequence* | BA | 32.3077 | 1.6062 |
| *Condition* | Condition A:no observater | 30.7433 | 1.5934 |
| *Condition* | Conidition B multiple observer | 34.4992 | 1.5934 |

| *Differences of Least Squares Means* | | | | | | |
| --- | --- | --- | --- | --- | --- | --- |
| *Effect* | *Difference* | *Differences of Least Squares Means* | *Standard Error* | *DF* | *t Value* | *Pr > \|t\|* |
| *sequence* | AB -BA | 0.6271 | 2.3444 | 47 | 0.27 | 0.7903 |
| *Condition* | Condition A: no oberver - Conidition B : multiple observer | -3.7559 | 2.1585 | 47 | -1.74 | 0.0884 |

**2.1 Outcome 3 : Relative change in HR (percentage)**

| *Type 3 Tests of Fixed Effects* | | | | |
| --- | --- | --- | --- | --- |
| *Effect* | *Num DF* | *Den DF* | *F Value* | *Pr > F* |
| *sequence* | 1 | 44.2 | 0.36 | 0.5508 |
| *Condition* | 1 | 43.6 | 29.53 | <.0001 |

| *Least Squares Means* | | | |
| --- | --- | --- | --- |
| *Effet* | *categorie* | *Estimate* | *Standard Error* |
| *sequence* | AB | 35.05 | 2.06 |
| *sequence* | BA | 35.78 | 1.90 |
| *Condition* | Condition A: sans observateur | 31.42 | 1.62 |
| *Condition* | Conidition B avec observateur | 39.40 | 1.63 |

| *Differences of Least Squares Means* | | | | | | |
| --- | --- | --- | --- | --- | --- | --- |
| *Effect* | *Difference* | *Differences of Least Squares Means* | *Standard Error* | *DF* | *t Value* | *Pr > \|t\|* |
| *sequence* | AB -BA | -2.50 | 4.16 | 44.2 | -0.6 | 0.5508 |
| *Condition* | Condition A: sans observateur - Conidition B avec observateur | -11.61 | 2.14 | 43.6 | -5.43 | <.0001 |

**Table2. Categorical outcomes**

| **Outcome** | **Condition A: no observer** | | | **Condition B: with observer** | | | **pvalue*** |
| --- | --- | --- | --- | --- | --- | --- | --- |
|  | **N** | **frequency** | **%** | **N** | **frequency** | **%** |  |
| 1st trial | 49 | 31 | 63.27 | 49 | 29 | 59.18 | 0.8318 |
| Success rate | 49 | 39 | 79.59 | 49 | 32 | 65.31 | 0.1435 |
| *McNemar test |  |  |  |  |  |  |  |
